# Empowering Internal Medicine Residents Through Health Policy: A Simple Approach to Inspire and Teach Action

**DOI:** 10.7759/cureus.69198

**Published:** 2024-09-11

**Authors:** Robin Reister, Brandon Altillo

**Affiliations:** 1 Internal Medicine, Dell Medical School, Austin, USA

**Keywords:** advocacy education, graduate medical education (gme), health policy making, internal medicine residency, systems based practice

## Abstract

Background

Most physicians receive little or no teaching about health policy during residency, despite accreditation organizations emphasizing the need to do so. Lack of time, expert faculty, and institutional financial support can be barriers to implementing a health policy experience.

Aim

Our goal was to educate internal medicine residents about the legislative process, improve their ability to engage with lawmakers and inspire them to effect policy change in their role as physicians. We aimed to impart a basic knowledge of how health policy decisions are made and give residents hands-on experience in speaking to lawmakers without requiring significant faculty or resident time.

Settings

Residents attended an hour-long classroom presentation at our medical school. A portion of them attended a lobby day with the Texas Medical Association (TMA). This consisted of introductory training at the TMA building and then a meeting with individual legislators at the Capitol.

Participants

Sixty-three internal medicine residents received the classroom presentation. Thirteen residents attended the lobby day, ten of whom were in the primary care track, and three were residents in the categorical program who expressed interest and had scheduled availability. Three Dell Medical School faculty members also attended the lobby day.

Program description

Residents attended a one-hour classroom presentation about the legislative process, current health policy proposals, and local organizations and their policy priorities. Residents then participated in a lobby day with the Texas Medical Association (TMA) in the spring of 2023, which consisted of an introductory session with an issue briefing and training sessions. Residents were then separated into groups based on their zip code, guided in how to speak on behalf of their experiences, and met with their local lawmakers.

Program evaluation

We administered a pre- and post-experience survey. Only 16.7% had ever met with a lawmaker, but they highly rated the importance of engaging in health policy (mean±SD, 4.42±0.79). The experience significantly increased from pre- to post-experience their ability to contact their lawmaker (2.75 to 3.89), confidence in their ability to discuss policy with a lawmaker (2.33 to 3.56), and their sense of empowerment that they have a voice that can influence policy (2.92 to 4.22).

Discussion

Internal medicine residents view engagement with health policy as an important experience during training. By utilizing existing resources in our institution combined with an existing lobbying opportunity, we were able to improve resident confidence and abilities to engage in policymaking without substantial financial or time costs.

## Introduction

Recent policy decisions at the federal and state levels have had major effects on the lives of our patients and our practice as physicians. Evidence-based medicine is known to influence policy, and policy in turn can have significant effects on the health of our population [1,2]. For example, there has been an increase in infant and neonatal deaths associated with Texas’ 2021 ban on abortion in early pregnancy [3]. As generalists, we bear witness to the effects of health policies on our patient’s lives and health and can be forced to practice medicine in ways that go against the best evidence-based practice. This experience, combined with our position in a respected profession, puts us in one of the best positions to have a voice in health policy decisions. Despite this, physicians often have less civic engagement than the general population [4].

The Liaison Committee on Medical Education requires that medical school curricula include “The impact of disparities in health care on all populations and approaches to reduce health care inequities” [5]. Similarly, the 2020 Accreditation Council for Graduate Medical Education (ACGME) Internal Medicine Core competencies include systems-based practice: physician role in health care systems. To achieve Level 5 mastery, a resident must be “actively engaged in influencing health policy through advocacy activities at the local, regional, or national level" [3]. Despite these benchmarks, physicians have noted significant deficiencies in the health policy education that they receive in their training [6].

At the Dell Medical School Internal Medicine Residency Program, we have an emphasis on health equity and improving the healthcare landscape. We have incorporated “Health Equity Academic Half Days," which cover topics such as structural inequalities, bias in healthcare, and how social determinants affect health outcomes. Our residents reported that beyond learning about the challenges that our patients face, they had a desire for more opportunities for action. Austin is the capital of Texas, and our proximity to the Capitol building provided an excellent opportunity to learn how to take action at the place where policy decisions are made.

Teaching health policy can require comprehensive scheduling of multiple experts and hours of devoted classroom time. We recognized the importance of teaching health policy, but we did not have dedicated faculty time to create and teach a curriculum. Our aim was to offer a fundamental understanding of health policy decisions and practical experience in communicating with lawmakers, all without placing extensive demands on faculty or residents' time. We therefore utilized the resources that already existed at our medical school and in our community: our medical school’s health policy expert and the Texas Medical Association’s lobby days.

We structured our health policy experience with the following objectives: by the end of the activity, learners will be able to explain the Texas legislative process, identify key topics for the current legislative session and where various medical organizations stand on those topics, express increased confidence in their ability to talk to lawmakers about health policy and apply the experience to their careers in envisioning a future of involvement in health policy.

## Materials and methods

Our health policy experience was executed in spring 2023 in the following settings: a classroom presentation by the Dell Medical School Senior Policy Director, an introductory session at the Texas Medical Association building, and meeting with and lobbying legislators at their offices at the Texas Capitol.

The experience was developed for internal medicine residents at the Dell Medical School, which is an academic program at the University of Texas at Austin.

Sixty-three internal medicine residents received the classroom presentation. Thirteen internal medicine residents and three internal medicine faculty participated in the lobby day.

All Dell Internal Medicine residents received the classroom presentation during their Health Equity Academic Half Day. The one-hour presentation was created by the Dell Medical School Senior Policy Director and was designed to be relevant to physicians in training with little or no experience in health policy. It consisted of four components: the Texas legislative process, key topics for the current legislative session, UT Austin/Dell med policies on political activities, and advocacy. The portion on the Texas legislative process included timing of legislation, dates of interest, budget details and passage, and makeup of the House and Senate. The key topics portion included major issues and then specific health issues likely to be addressed, including abortion, transgender care, Medicaid, the workforce, and mental health.

For the lobbying portion, we hoped that by giving residents the practical experience of speaking to a lawmaker even one time, they would feel motivated and more confident in civic engagement in the future. We chose to partner with the Texas Medical Association (TMA) given their wealth of experience and expertise in the process. During each legislative session, TMA organizes “First Tuesdays at the Capitol,” which serves as a call for all doctors, trainees, and students throughout Texas to participate in advocacy for the priorities TMA has agreed on.

Thirteen internal medicine residents and three internal medicine faculty participated in First Tuesdays at the Capitol. We were unable to arrange coverage of resident duties for all residents, so for our first year of the experience, we prioritized residents in the primary care track and included categorical residents who expressed interest in participating. One of the goals of the primary care track is to train leaders to address social determinants of health, and experience in health policy helps to achieve this goal. The faculty that participated volunteered to join during their non-clinical time because they expressed interest in getting involved with and teaching health policy; they did not have a specific health policy or advocacy role at the medical school. We thought it was important that faculty participated to model engagement in health policy while also gaining experience so that they can mentor residents in the future. There were four different lobby days available; residents only attended one of the days.

The introductory session was at the Texas Medical Association Building in downtown Austin, and our residents participated with hundreds of other physicians and trainees from throughout Texas. The First Tuesday schedule included a welcome from TMA leadership, a presentation from a Texas legislator (Senator or Representative) discussing their health policy goals and achievements, an issue briefing from professional lobbyists, and a Grassroots Advocacy 101 session.

Participants were then separated into groups based on their zip code to meet with their local representative. Each group had a physician leader with several years of experience who helped to guide the other physicians. The groups had four scheduled meetings with different lawmakers for about thirty minutes each. Residents were encouraged to speak directly to legislators and were given tips on how to be concise and effective when speaking of their particular experiences and needs. Typically, each group met with lawmakers from different parties (ex., two Democrats and two Republicans).

To evaluate the health policy experience, we administered a pre-first Tuesday and post-first Tuesday survey for the residents who participated in the lobby day (N=13). The survey was designed to assess prior knowledge and experience with engaging in health policy, interest and perceived importance, and confidence in engaging in health policy. Items were scored on a five-point Likert scale. Differences in pre- and post-survey scores were assessed using one-tailed unpaired t-tests. We included a section for comments and feedback in the post-survey.

## Results

Twelve residents (92%) completed the pre-survey, and nine (69%) completed the post-survey. Only 2/12 (16.7%) had ever met with a lawmaker before the experience.

**Figure 1:**
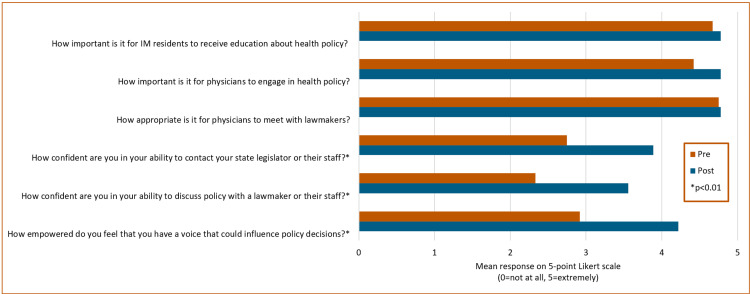
Pre/post-survey results Mean response on a five-point Likert scale; 0=not at all, 5=extremely. N=12 pre, N=9 post. *p<0.01 IM: internal medicine

Figure 1 summarizes the pre- and post-survey responses. Both pre-and post-First Tuesday, residents highly rated the importance of health policy education in internal medicine residency (mean pre=4.67, post=4.78, p=0.30) and the importance of physicians engaging in health policy (pre=4.42, post=4.78, p=0.10). They also largely agreed that it is appropriate for physicians to meet with lawmakers (pre=4.75, post=4.78, p=0.44).

**Figure 2:**
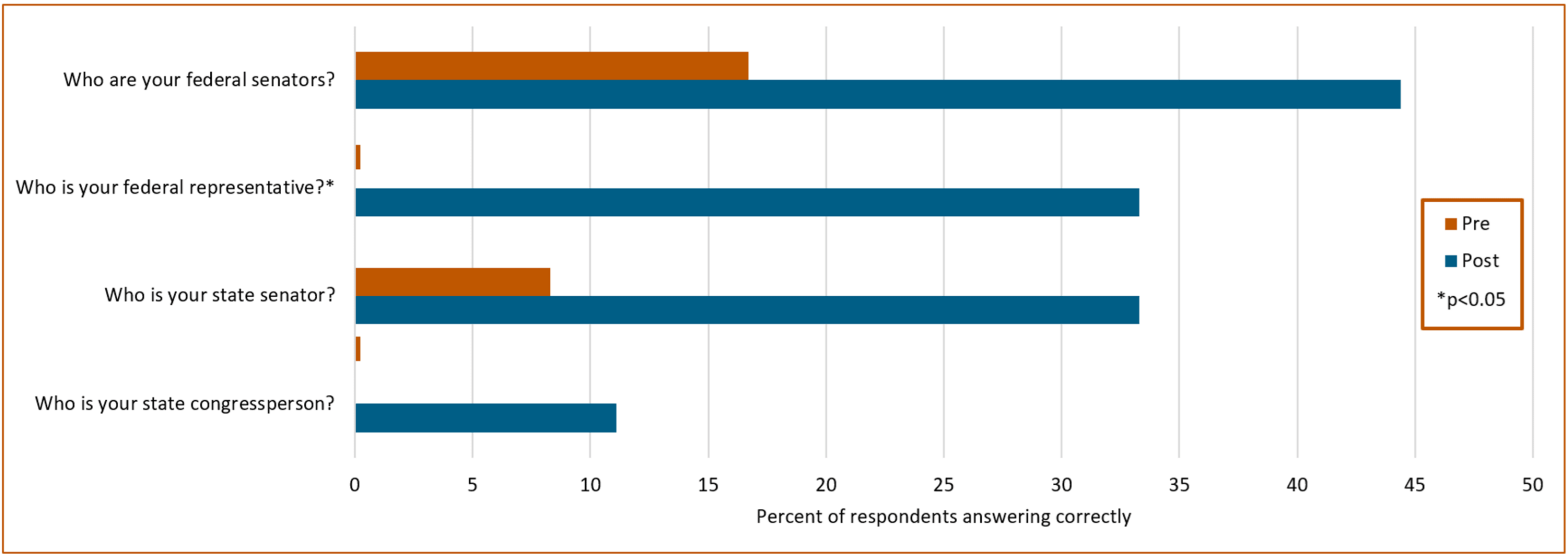
Pre/post-survey results Percent of respondents who correctly answered each question. N=12 pre, N=9 post. *p<0.05

The post-survey (Figure 2) showed a statistically significant increase in residents’ confidence in their ability to contact their lawmaker (pre=2.75, post=3.89, p<0.001), their confidence in their ability to discuss policy with a lawmaker (pre=2.33, post=3.56, p=0.003), and their sense of empowerment that they have a voice that can influence policy (pre=2.92, post=4.22, p<0.001).

We also assessed residents’ knowledge of their federal and state legislative representatives pre- and post-First Tuesday (Figure 2). There were trends toward improvement in the share of respondents who could name their legislators correctly based on their zip code, but only the change in the knowledge of the federal representative was statistically significant (state senator, pre=1/12 (8.3%), post=3/9 (33.3%), p=0.10; state representative, pre=0/12 (0%), post=1/9 (11.1%), p=0.17; federal senators, pre=2/12 (16.7%), post=4/9 (44.4%), p=0.077; federal representative, pre=0/12 (0%), post=3/9 (33.3%), p=0.04).

Comments included: “excellent education on health policy, advocacy, and organized medicine," “amazing experience, recommend heavily," “10 out of 10, I finally felt like we were doing something.”

## Discussion

Our program evaluation showed that internal medicine residents rate their engagement in health policy as important and that our experience increased their confidence and empowerment. Combining a resident-specific educational session and local faculty mentorship with an existing advocacy opportunity allowed us to show our residents an example of well-organized lobbying and advocacy without a significant time commitment from the faculty.

Only two residents in our evaluation had ever met with a lawmaker before the experience, which underscores the importance of even a one-time meeting such as First Tuesday to overcome the challenge of lack of experience in engaging with elected officials. The first three Likert questions showed no difference pre- and post-First Tuesday, largely because they rated importance and appropriateness highly in the pre-survey. This highlights the anecdotal reports that our residents were enthusiastic about and willing to engage, and is consistent with prior reports that doctors in training feel that knowledge of health policy is important to their career [7]. The last three Likert questions showed significant increases in feelings of empowerment and confidence in contacting and engaging with policymakers, showing that even a one-time experience at the Capitol can have an impact. 

Although there was an increase in the knowledge of their state representative, all four knowledge questions about the names of representatives and senators were less than 50% correct. The names of lawmakers were not emphasized in the experience, so this is not surprising. Furthermore, we believe that it is more important that they have the tools and confidence to find their representative and know how to contact them than to remember their name. A better test of knowledge could have been to ask questions about the legislative process that they were taught in the classroom portion of the experience. We plan to refine our evaluation tool in future iterations of the experience.

Often, health policy trainings are done at the medical student level rather than the residency level, perhaps because of greater flexibility in scheduling, a larger pool of learners to find ones with an interest [8], and often more resources [9]. Cornell Medical School implemented a required, in-depth medical student curriculum that combined expert-led lectures with field assignments and dedicated two weeks to the clerkship [10]. Although this curriculum was well-received, often residency programs would find difficulty committing two weeks away from clinical duties, and not all institutions have access to health policy experts to lead lectures.

Some internal medicine residency programs have had success implementing a health policy curriculum, but most that are described benefit from substantial resources. The Cambridge Health Alliance Internal Medicine program implemented a required, yearlong longitudinal curriculum [11]. This program had two-course directors with 10% of their full-time equivalent dedicated to this and also other program financial support, making it less generalizable to many programs. Internal medicine residents at Columbia University participated in a week-long curriculum, a time commitment that is smaller than some, but that program also benefited from partial funding and expert faculty [12]. Greysen et al. describe a unique course at George Washington University that combined interested residents from 14 different specialties and included small group seminars with policy experts and many site visits to policymakers in Washington, D.C. [13]. This program had the advantage of 45-50 individuals to deliver didactic material or lead groups, with many of them from the George Washington Department of Health Policy.

Our experience is applicable to other programs that want to offer a health policy experience but do not have access to additional faculty time, financial support, or substantial resident time away from other clinical duties. The Dell Medical School Senior Policy Director was eager to create a classroom presentation, and the Texas Medical Association was enthusiastic about welcoming more residents to participate in their lobby day. These existing resources allowed us to implement this experience with minimal effort while accomplishing our goal of experiencing and understanding the physician's role in policymaking. We utilized existing classroom time and were only one day away from clinical duties for the residents who met with lawmakers.

One downside of the structure of this experience is the need to stay within the bounds of the TMA’s legislative priorities. Many of our residents expressed a desire to lobby more strongly for or against certain policies. As our faculty and residents gain more experience in lobbying, we could encourage them to lobby independently in their own time for healthcare policy priorities that are most important to them.

Limitations of this evaluation include the small sample size and attrition in response rates between the pre-and post-surveys. Additionally, the responses of these residents may not be representative of internal medicine residents more broadly since the categorical residents self-selected into the experience, and the primary care track residents may be more likely to be interested in health policy and advocacy. It would also be useful to include residents from specialties other than internal medicine. 

## Conclusions

Our innovation serves as a successful example of health policy in action, requiring minimal faculty time or experience. We plan to continue this experience and enhance it by introducing specific skills in the didactic portion, such as op-ed writing, media, and social media interaction, data as policy, and creating an elevator pitch. We are hoping that the more hands-on experience our learners gain, the more likely they will be to stay active in health policy work.

Legislative decisions will continue to significantly impact our practice of medicine and the lives of our patients. Topics such as gender-affirming care, reproductive access, funding for mental health, expansion of Medicaid, and workforce development are likely to be debated in upcoming sessions, and physicians will have the opportunity to share their expert opinion and firsthand experience. It is essential to educate medical residents about the impact of health policy on themselves and their patients, as well as to empower them to influence the pivotal decisions made by lawmakers.
